# Assessing health systems’ capacities to provide post-abortion care: insights from seven low- and middle-income countries

**DOI:** 10.7189/jogh.15.04020

**Published:** 2025-01-10

**Authors:** Sahar Raza, Rajon Banik, Syed Toukir Ahmed Noor, Esrat Jahan, Abu Sayeed, Nafisa Huq, Shams El Arifeen, Anisuddin Ahmed, Ahmed Ehsanur Rahman

**Affiliations:** 1 Maternal and Child Health Division, International Centre for Diarrhoeal Disease Research, Bangladesh (icddr,b), Dhaka, Bangladesh.; 2 Independent University, Bangladesh (IUB), Dhaka, Bangladesh.

## Abstract

**Background:**

Abortion-related complications significantly contribute to maternal morbidity and mortality globally. Post-abortion care (PAC) services are essential to safeguarding women’s rights by substantially mitigating the health risks associated with abortions – a step which is fundamental to achieving reproductive and maternal health-related Sustainable Development Goals.

**Methods:**

We conducted a secondary analysis of data from the nationally representative Service Provision Assessment (SPA) surveys conducted between 2015 and 2024 across three regions in seven low- and middle-income countries: Afghanistan, Bangladesh, Nepal, the Democratic Republic of Congo (DRC), Ethiopia, Senegal, and Haiti. We included 2951 primary facilities and 473 referral facilities offering normal delivery services. We used PAC signal functions to report capacity to provide basic and comprehensive PAC services in primary and referral facilities, respectively.

**Results:**

Of all primary facilities offering normal delivery services, 50% in Afghanistan, 1% in Bangladesh, 8% in Nepal, 5% in DRC, 34% in Ethiopia, 38% in Senegal, and 19% in Haiti had the capacity to provide basic PAC services. Of the referral facilities, 47% in Afghanistan, 16% in Bangladesh, 50% in Nepal, 52% in DRC, 75% in Ethiopia, 46% in Senegal, and 32% in Haiti had the capacity to provide comprehensive PAC services. Primary facilities in Bangladesh, DRC, and Nepal had critical gaps in referral, ie, effective communication with referral centres and availability of a functional vehicle for emergency transportation. In referral facilities, 74% in Bangladesh and 59% in Nepal had the provision of blood transfusion. In terms of basic PAC services in primary facilities, the capacity of Senegal (from 16% in 2015 to 38% in 2019; *P* = 0.001) and Haiti (from 12% in 2013 to 19% in 2018; *P* = 0.007) increased, but the capacity of Bangladesh decreased (from 4% in 2014 to 1% in 2017; *P* = 0.016) over time.

**Conclusions:**

There are substantial gaps in the capacity to provide basic and comprehensive PAC services in the selected countries. Investing in primary healthcare and improving communication and transportation should be the priority for enhancing basic PAC services, while strengthening referral hospitals to effectively handle emergencies and conduct major surgeries could significantly bolster their capacity to provide comprehensive PAC services.

Abortion is a major public health concern globally [1]. According to the World Health Organization (WHO), approximately 73 million induced abortions occur each year, with 45% being classified as unsafe [2]. Complications related to abortion, particularly unsafe abortions, contribute to more than 16 thousand maternal deaths and 1.1 million disability-adjusted life years (DALYs) every year [3]. Approximately 99% of these abortion-related deaths and disabilities happen in low- and middle-income countries (LMICs) [3].

Post-abortion care (PAC) encompasses a comprehensive package of services tailored to meet the needs of women who have undergone spontaneous or induced abortions. As a fundamental human right, it is considered an essential health service [4,5]. This comprehensive approach includes emergency treatment for abortion-related complications, such as haemorrhage, infections, or retained products of conception, as curative elements. It also incorporates counselling on family planning methods as a preventive component, emphasising the importance of birth spacing and contraceptive use to prevent unintended pregnancies and subsequent abortions [6]. Mid-level health service providers that offer normal delivery services in health facilities can effectively administer PAC [1,7], which, by ensuring safe, timely, and respectful care, functions as a cost-effective strategy to mitigating preventable morbidity and mortality associated with abortion-related complications [8]. Unfortunately, in LMICs, only 6% of women requiring PAC services following unsafe abortions actually receive them [9].

A comprehensive understanding of healthcare facility capacity, including their availability, readiness, and functionality, could uncover gaps and guide targeted interventions to enhance coverage and quality of PAC services [10]. Regrettably, limited evidence exists regarding the status and capacities of healthcare facilities to provide PAC services, particularly in LMICs and settings with limited resources. An earlier study conducted using data from 2007 to 2015 highlighted gaps in PAC service provision across 10 LMICs, including Bangladesh, Haiti, Kenya, Malawi, Namibia, Nepal, Rwanda, Senegal, Tanzania, and Uganda [11]. Yet the establishment of the Sustainable Development Goals (SDGs) and the coronavirus disease 2019 (COVID-19) pandemic affected the health systems and essential services worldwide – including PAC services [12–15]. An in-depth understanding PAC capacity in the post-pandemic era is crucial for adapting services to the evolving context. Therefore, in this study, we aimed to understand the current capacity of health systems to provide PAC services and report the change in PAC capacity in selected LMICs over the time period of 2015–24.

## METHODS

### Data sources

We conducted a secondary analysis of data from Service Provision Assessment (SPA) surveys carried out by the Demographic and Health Surveys programme. The SPA is a cross-sectional assessment of health facilities on the general availability, readiness and functionality of a range of services, such as basic amenities, commodities, family planning, antenatal care, delivery and newborn care, post-natal care, child care, and diagnostics. It is conducted with a nationally representative sample, including all major types of health facilities in a country [16].

We identified 12 surveys conducted between 2015 and 2024 across seven countries (Figure S1 in the **Online Supplementary Document**). For countries with multiple rounds of SPA within this period, we selected the most recent round of SPA. Finally, we included data from surveys in Afghanistan (2018–19), Bangladesh (2017), Nepal (2021), DRC (2017–18), Ethiopia (2021–22), Senegal (2019), and Haiti (2017–18) for analysis. Details regarding the SPA methodology, sampling, data collection tools and data collection procedure are available elsewhere [17].

Facilities providing delivery services should theoretically be able to provide post-abortion care [11]. We therefore included all health facilities with completed interviews that reported offering delivery services in our analysis: 112 (out of 142) from Afghanistan, 358 (out of 1524) from Bangladesh, 1328 (out of 1380) from the DRC, 217 (out of 1158) from Ethiopia, 361 (out of 1007) from Haiti, 805 (out of 1576) from Nepal, and 243 (out of 425) from Senegal (Table S1 in the **Online Supplementary Document**).

### Signal functions

We adopted the signal functions identified by Campbell and colleagues [10] in reporting the capacities of public health facilities to provide PAC services; they identified eight signal functions to evaluate the capacity of health facilities in delivering basic PAC services and nine signal functions for assessing comprehensive PAC service (**Table 1**; Table S2 in the **Online Supplementary Document**).

**Table 1:** Signal functions for providing basic and comprehensive PAC services and their operational definitions

		Basic PAC services			Comprehensive PAC services		
**Signal function**	**Operational definition**	**Most restrictive**	**Less restrictive**	**Least restrictive**	**Most restrictive**	**Less restrictive**	**Least restrictive**
Removal of retained products of conception (basic signal function 1 and comprehensive signal function 1)	Availability of a surgical apparatus (vacuum aspirator or dilation & curettage (D&C) kit reported functioning) or misoprostol (observed and valid) in the facility at the time of the survey.	Yes	Yes	Yes	Yes	Yes	Yes
Administer parenteral antibiotics (basic signal function 2 and comprehensive signal function 2)	Providers reported ever having administered antibiotics parenterally intravenously or intramuscularly as part of their work in the facility at the time of the survey.	Yes	Yes	Yes	Yes	Yes	Yes
Administer parenteral uterotonics (basic signal function 3 and comprehensive signal function 3)	Providers reported the availability of injectable oxytocin, ergometrine, or other uterotonics (injectable uterotonic (oxytocin) or methyl ergometrine injection) in the facility at the time of the survey.	Yes	Yes	Yes	Yes	Yes	Yes
Administer intravenous fluids (basic signal function 4 and comprehensive signal function 4)	Availability of any of the following intravenous fluids (observed functioning/ reported available): normal saline (500 ml or 1000 ml), Ringer's lactate solution (1000 ml), dextrose in normal saline 0.9% (500 ml or 1000 ml) in the facility at the time of the survey.	Yes	Yes	Yes	Yes	Yes	Yes
Modern short-acting family planning methods available at the time of the survey (basic signal function 5 and comprehensive signal function 5)	At least one method was reported available in the facility: combined oral pill, progesterone-only pill, combined injectable, progestin-only injectable, or male condoms in stock and valid, or report providing counselling on natural methods at the time of the survey.	Yes	Yes	Yes	Yes	Yes	Yes
Capacity to communicate with referral facilities (basic signal function 6)	Availability of a landline or cellular phone, or a private cellular phone reimbursed by the facility or higher authority, observed and functioning at the time of the survey.	Yes	Yes	No	No	No	No
A vehicle with fuel for referral (basic signal function 7)	The facility has a functional ambulance or other vehicle with fuel for emergency transportation that is stationed at and operates from the facility (observed), or the facility has access to an ambulance or other vehicle (reported) for emergency transportation stationed at or operating from another facility with fuel at the time of the survey.	Yes	Yes	No	No	No	No
Staff capable of undertaking vaginal deliveries available 24 h per day, seven days per week (basic signal function 8)	A person skilled in conducting deliveries (medical specialists, medical officers, nurses, family welfare visitors, and midwives) is present at the facility today or on call at all times (24 h a day), including weekends, to provide care (reported) at the time of the survey.	Yes	No	No	No	No	No
Administer a blood transfusion (comprehensive signal function 6)	Blood transfusion has been conducted (reported) in this facility in an obstetric context (ie, for maternal care) within the past three months.	No	No	No	Yes	Yes	Yes
Undertake major abdominal surgery (proxied by provision of caesarean section) (comprehensive signal function 7)	The facility offers caesarean sections and/or has provided caesarean sections within the past three months (reported).	No	No	No	Yes	Yes	No
Provide at least one long-acting, reversible family planning method or permanent method at the time of the survey (comprehensive signal function 8)	At the time of the survey, at least one method was available: intrauterine device (observed available), implant (observed in stock and valid), or report providing male or female sterilization (without regard to time period).	No	No	Yes*	Yes	Yes	Yes
Has staff capable of doing caesarean sections on duty or who are on call 24 h per day, seven days per week (comprehensive signal function 9)	The presence of a health worker (includes medical specialists, obstetricians, surgeons, or other trained personnel) who can perform caesarean deliveries (sections) at the facility or is on call 24 h a day, including weekends and public holidays (reported).	No	No	No	Yes	No	No

PAC – post-abortion care

*Signal function is newly included in the less restrictive composite indicators of basic PAC.

The basic PAC signal functions comprise commodities or services to ensure facilities can provide immediate support for less complicated cases following an abortion and referring severe cases, which include:

1. the removal of retained products of conception;
2. the administration of parenteral antibiotics;
3. the administration of parenteral uterotonics;
4. the administration of intravenous fluids;
5. the provision of at least one modern, short-acting family planning method at the time of the survey;
6. communication with referral facilities;
7. having a vehicle with fuel to transport patients needing referral;
8. having staff capable of undertaking normal deliveries on duty or who are on call for 24 hours per day, seven days per week.

Comprehensive PAC signal functions comprise commodities or services for all basic functions and speciality care essential for managing complications and include:

1. the removal of retained products of conception;
2. the administration of parenteral antibiotics;
3. the administration of parenteral uterotonics;
4. the administration of intravenous fluids;
5. the provision of at least one modern, short-acting family planning method at the time of the survey;
6. the administration of blood transfusions
7. the capacity to undertake major abdominal surgery (proxied by the provision of caesarean section)
8. the provision of at least one long-acting reversible family planning method (intrauterine devices or hormonal implants) or permanent method (i.e., female and male sterilisation) at the time of the survey;
9. having staff capable of doing caesarean sections on duty or who are on call for 24 hours per day, seven days per week.

Five signal functions (1–5) are common to both basic and comprehensive PAC services. Besides the first five signal functions, comprehensive PAC services included four more. Basic PAC services (all basic signal functions) should be available in all facilities offering childbirth services, while comprehensive PAC services (all comprehensive signal functions) should be available in all referral facilities.

### Statistical analysis

We imported the data from each country into Stata, version 17.0 (StataCorp LLC., College Station, Texas, USA) for cleaning and analysis. We adjusted the number of facilities of each type to reflect their proportional contribution to the total number of facilities, aligning with the overall distribution of health facilities in each respective country. We then organised the results based on the years of the surveys and the type of facility (primary and referral) according to each country (Table S3 in the **Online Supplementary Document**).

We initially reported the proportion of facilities (both primary and referral) that performed each of the signal functions to provide basic and comprehensive PAC services, after which we assessed their capacity to provide basic PAC services with a composite indicator adopting three definitions: most restrictive, less restrictive and least restrictive [10,11,17–19]. The most restrictive indicator included all eight basic signal functions. The less restrictive composite indicator included all basic signal functions except staff availability (24/7) to perform normal deliveries (basic signal function 8). The least restrictive composite indicator included all basic signal functions except staff availability (24/7) to perform normal deliveries (basic signal function 8), capacity to communicate with referral facilities (basic signal function 6), and vehicles with fuel to transport patients needing referral (basic signal function 7). This indicator also includes the availability of long-acting or permanent family planning methods (comprehensive signal function 8).

We also used three tiers of indicators to assess the facilities' capacity to provide comprehensive PAC services. The most restrictive indicator considered all aspects necessary for delivering PAC services, including all nine comprehensive signal functions. Less restrictive indicator excluded personnel who could deliver services at the primary facility level ie, staff availability (24/7) and those qualified to perform caesarean sections at the referral level (comprehensive signal function 9). Least restrictive indicator approach went further by omitting the requirements for the ability to perform major abdominal surgeries (comprehensive signal function 7). This tiered approach helped us evaluate the facilities' capacities in a more nuanced way.

We reported the current status and capacity based on the latest available data (2015–24). Additionally, we analysed the trend in the capacity to provide basic PAC services and comprehensive PAC services for each country (based on the availability of data) over time. We used proportion tests to report any significant changes. All proportions were reported with 95% confidence intervals (CIs), and the significance level was set at *P* < 0.05.

## RESULTS

### Status of signal functions to provide PAC services

Approximately 84% of primary facilities in Afghanistan had a functioning surgical apparatus (either a vacuum aspirator or a D&C kit) at the time of the survey for removing retained products of conception (basic signal function 1), in contrast to only 39% in Bangladesh and 40% in Nepal. Furthermore, more than three-fourths of the facilities in Afghanistan (84%), Bangladesh (98%), Ethiopia (94%), and Haiti (84%) had ever administered parenteral antibiotics (basic signal function 2). However, this figure was significantly lower in Nepal (31%). Regarding family planning, over four-fifths of the facilities in Afghanistan (80%), Bangladesh (98%), Ethiopia (99%), Senegal (92%), and Haiti (82%) had at least one modern, short-acting family planning method available or provided counselling on natural methods at the time of the survey (basic signal function 5). This proportion was 59% in Nepal and 69% in the DRC. In terms of communication infrastructure, nearly all facilities in Afghanistan (93%) and three-fourths of the facilities in Senegal (77%) and Haiti (75%) had a functioning device to communicate with referral facilities (basic signal function 6). However, only 8% of facilities in Bangladesh and 18% in Nepal had this capacity. Regarding patient transportation, approximately 91% of facilities in Afghanistan and 88% in Ethiopia had a functional vehicle with fuel or access to a vehicle for referring patients to higher-level facilities (basic signal function 7), compared to only 13% in Bangladesh and 16% in the DRC. Finally, concerning staff capacity (basic signal function 8), more than 90% of facilities offering childbirth services in Afghanistan, DRC, Ethiopia, and Senegal had personnel trained in conducting vaginal births, available 24 hours a day, seven days a week. In Bangladesh and Nepal, this proportion was close to 50% (**Table 2**).

**Table 2:** Status of signal functions to provide basic PAC services among primary facilities offering delivery services in seven LMICs, 2015–24

	South Asia			Sub-Saharan Africa			Caribbean
**Signal functions**	**Afghanistan (n = 67)**	**Bangladesh (n = 266)**	**Nepal (n = 702)**	**Democratic Republic of Congo (n = 1196)**	**Ethiopia (n = 193)**	**Senegal (n = 231)**	**Haiti (n = 296)**
Basic signal function 1: removal of retained products of conception	84%	39%	40%	63%	86%	74%	71%
Basic signal function 2: administer parenteral antibiotics	84%	98%	31%	79%	94%	77%	84%
Basic signal function 3: administer parenteral uterotonics	94%	50%	56%	96%	99%	95%	94%
Basic signal function 4: administer intravenous fluids	100%	45%	58%	81%	97%	94%	96%
Basic signal function 5: provision of at least one modern, short-acting family planning method at the time of the survey	80%	98%	59%	69%	99%	95%	82%
Basic signal function 6: capacity to communicate with referral facilities	93%	8%	18%	51%	48%	77%	75%
Basic signal function 7: vehicle with fuel for referral	91%	13%	48%	16%	88%	70%	47%
Basic signal function 8: staff capable of undertaking vaginal deliveries available 24 h per day, seven days per week	95%	48%	58%	96%	93%	99%	82%

More than three-fourths of all referral facilities in all countries had a functioning surgical apparatus (either a vacuum aspirator or a D&C kit) at the time of the survey for removing retained products of conception (comprehensive signal function 1). It was almost universal in Afghanistan (97%) and Ethiopia (97%). Almost all referral facilities in all countries had ever administered parental antibiotics (comprehensive signal function 2), except in Nepal (approximately 69%). Injectable uterotonics (comprehensive signal function 2) were almost universally available in all countries, except in Nepal (75%). More than 90% of all referral facilities in the selected countries had intravenous fluids (comprehensive signal function 4) available on the day of the survey, except for those in Nepal (78%). Modern short-acting family planning methods (comprehensive signal function 5) were available in 90% of facilities in Afghanistan and 95% of facilities in Ethiopia. However, the availability was between 70% and 80% in Bangladesh, Nepal, and DRC. Haiti had the lowest availability at 59%. Almost all (98%) of the referral facilities in DRC performed blood transfusions (comprehensive signal function 6) in the past three months. The presence of this functionality ranged between 78% and 88% for the facilities in Afghanistan, Ethiopia, Senegal and Haiti, and was much lower in Bangladesh (54%) and Nepal (59%). The provision to undertake major abdominal surgery (comprehensive signal function 7), represented by the performance of a caesarean section in the past three months, varied across countries. Nepal and Bangladesh registered the lowest functionality at 58% and 65%, respectively, while more than 80% of the rest of the countries had around 80% or more capacity. More than 80% of the referral facilities in all countries had at least one long-acting method or permanent method of family planning (comprehensive signal function 8) available at the time of the survey, with the exception of Nepal (68%). Finally, approximately 80% or more of the referral facilities in all countries had 24/7 availability (on duty or on-call) of staff capable of performing caesarean sections (comprehensive signal function 9), except for Nepal and Bangladesh, which were at 57% and 61%, respectively (**Table 3**).

**Table 3:** Status of signal functions to provide comprehensive PAC services among referral facilities offering delivery services in seven LMICs, 2015–24

	South Asia			Sub-Saharan Africa			Caribbean
**Signal functions**	**Afghanistan (n = 45)**	**Bangladesh (n = 92)**	**Nepal (n = 132)**	**Democratic Republic of Congo (n = 24)**	**Ethiopia (n = 65)**	**Senegal (n = 103)**	**Haiti (n = 12)**
Comprehensive signal function 1: removal of retained products of conception	97%	75%	79%	82%	97%	87%	82%
Comprehensive signal function 2: administer parenteral antibiotics	92%	99%	69%	95%	99%	96%	100%
Comprehensive signal function 3: administer parenteral uterotonics	92%	97%	75%	99%	99%	100%	98%
Comprehensive signal function 4: administer intravenous fluids	100%	94%	78%	93%	96%	100%	100%
Comprehensive signal function 5: provision of at least one modern, short-acting family planning method at the time of the survey	90%	74%	70%	80%	95%	75%	59%
Comprehensive signal function 6: a blood transfusion	78%	54%	59%	98%	87%	79%	88%
Comprehensive signal function 7: undertake major abdominal surgery (proxied by provision of caesarean section)	92%	65%	58%	98%	92%	79%	86%
Comprehensive signal function 8: provided at least one long-acting, reversible family planning method or permanent method at time of the survey	82%	91%	68%	90%	98%	100%	84%
Comprehensive signal function 9: has staff capable of doing caesarean sections on duty or who are on call 24 h per day, seven days per week	90%	61%	57%	97%	91%	79%	81%

### Capacity to provide PAC services

**Figure 1:**
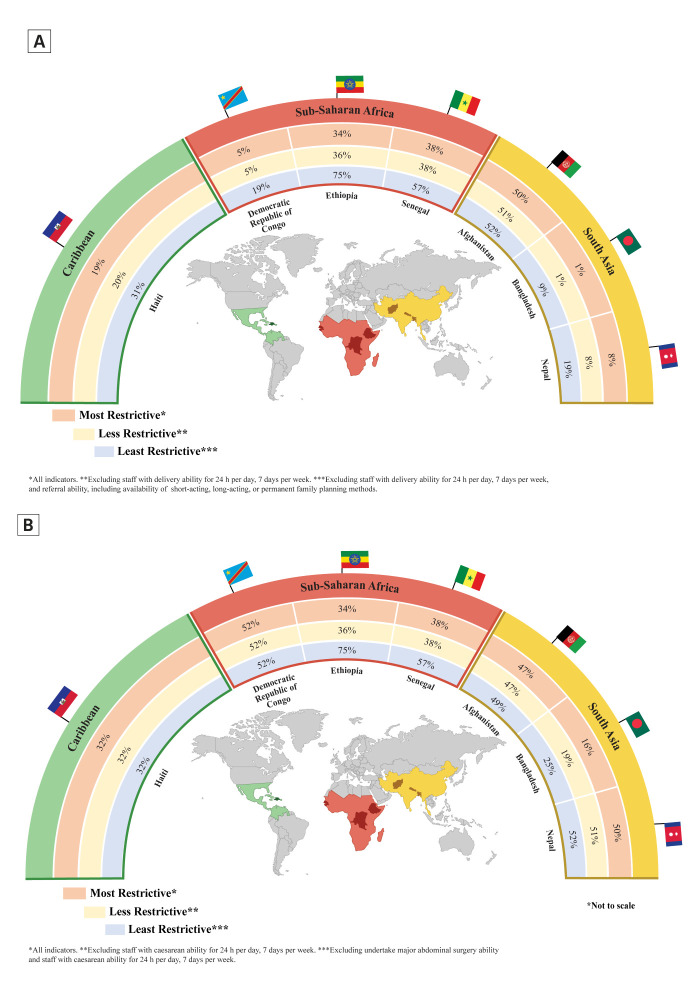
Capacity to provide basic PAC services across seven LMICs, 2015–24. **Panel A.** Primary facilities. **Panel B.** Comprehensive PAC services in referral facilities.

The capacity to provide basic PAC services (all eight signal functions) was the highest in Afghanistan (50%), followed by Senegal (38%), Ethiopia (34%), and Haiti (19%). Less than 10% of the primary facilities in Bangladesh (1%), DRC (5%), and Nepal (8%) had this capacity. When considering the less restrictive definition, the capacity almost remained unchanged across all countries. However, when considering the least restrictive definition, the capacity to provide basic PAC services was the highest in Ethiopia (75%), followed by Senegal (57%), Afghanistan (52%), and Haiti (31%), and was lowest in Bangladesh at 9% (**Figure 1**, Panel A).

Ethiopia had the highest capacity (75%) in providing comprehensive PAC (most restrictive composite indicator), followed by the DRC (52%) and Nepal (50%). Haiti, Senegal, and Afghanistan showed low to moderate capacity, ranging from 32% to 47%. Bangladesh had the lowest capacity to provide comprehensive PAC services (16%). The capacities remained relatively unchanged in all countries when reporting with the less restrictive definition, except for Bangladesh (19%). When considering the least restrictive definition, the capacity to provide comprehensive PAC was the highest in Ethiopia at 75% and the lowest in Bangladesh at 25% (**Figure 1**, Panel B). All estimates presented in the results section are provided with 95% CI in Tables S4 and S5 of the **Online Supplementary Document****.**

### Trend in capacity to provide PAC services

**Figure 2:**
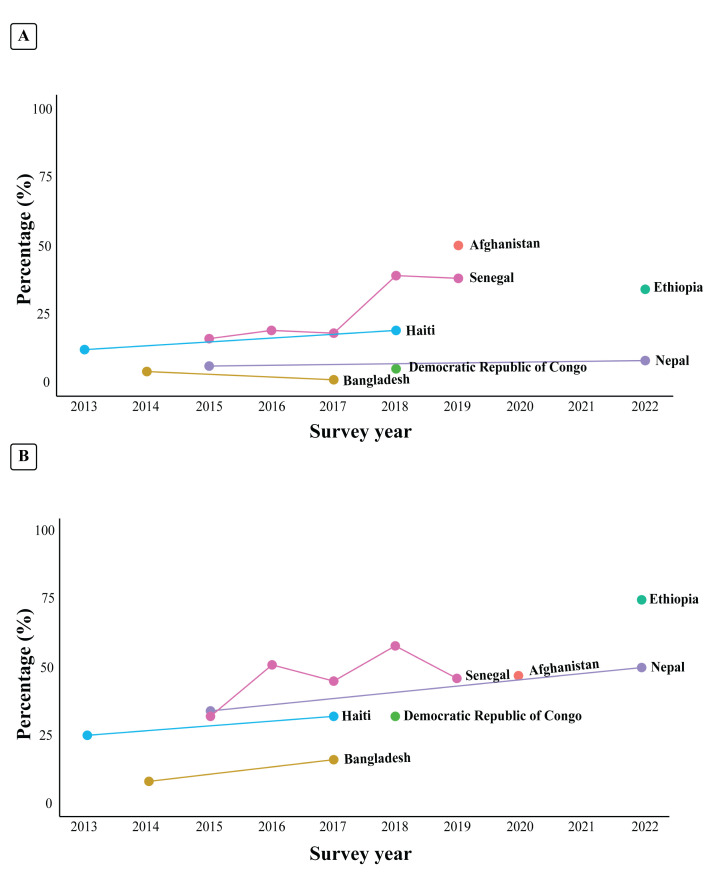
Trends in capacity to provide basic PAC services across seven LMICs. **Panel A.** Primary facilities. **Panel B.** Comprehensive PAC services in referral facilities.

In terms of basic PAC services in primary facilities (**Figure 2**; Table S6 in the **Online Supplementary Document**), the capacity of Senegal (from 16% in 2015 to 38% in 2019; *P* = 0.001) and Haiti (from 12% in 2013 to 19% in 2018; *P* = 0.007) increased over time. However, the capacity in Bangladesh decreased over time (from 4% in 2014 to 1% in 2017; *P* = 0.016).

## DISCUSSION

All health facilities offering childbirth services should have the capacity to offer PAC, regardless of the abortion method, who performed it, or whether it was permitted or not in that country. The aftermath of abortion can starkly amplify women's physical and mental complications and may lead to fatal consequences [2,20]. This is compounded by the existing inadequacies in healthcare systems and service delivery provisions, particularly concerning women's needs, and the pervasive stigma surrounding abortion [20–22]. We comprehensively examined health facilities based on a set of validated signal functions and reported the gaps in the capacity to provide basic and comprehensive PAC services in seven LMICs across sub-Saharan Africa, South Asia, and the Caribbean [10,11]. We also identified the variability and specific gaps in the provision of individual signal functions across these seven countries in primary and referral facilities separately. The comprehensive reporting of gaps in PAC services with nationally representative sample of health facilities in the post-COVID-19 era highlights the uniqueness, importance, and implication of this study for policy and programme planning. As we focussed only on facilities offering delivery services, it is worth noting that the proportion of facilities with basic and comprehensive PAC capability would have likely been lower if all types of facilities were included.

It is also worth noting that health systems in Afghanistan, Bangladesh, Nepal, the DRC, Ethiopia, Senegal, and Haiti differ widely due to sociopolitical, economic, and infrastructural factors, while the countries themselves differ by population, gross domestic product, health expenditure, and maternal mortality rates (Table S7 in the **Online Supplementary Document**). Political instability can disrupt funding [23] and hinder infrastructure development [24], while corruption diverts resources intended for healthcare, leading to shortages of medicines, supplies, and trained personnel [25,26]. Additionally, social inequality, often worsened by weak policies, limits access to care for marginalized populations, further straining health systems [27,28].

Primary care facilities are the first point of contact for availing essential services, including PAC. They are expected to provide basic preventive, promotive, and curative services to women after they experience abortion, and perform early stabilisation and appropriate referral for complicated cases [29,30]. Ensuring the availability, readiness, and functionality of such basic services in primary facilities is particularly important for populations residing in rural and urban areas with geographical inaccessibility or financial hardship [31]. Unfortunately, we found substantial gaps in the capacity to provide basic PAC services in the selected countries. Around half of the primary facilities in Afghanistan have the capacity to provide basic PAC, and even fewer (<10%) in Bangladesh, Nepal, and DRC. The overall capacity of these countries’ facilities to provide basic emergency obstetric care and curative care in these countries is also grossly inadequate and inappropriate [32–34]. These gaps highlight the apparent lack of prioritising not just PAC services but the overall primary healthcare in policy and programmes.

Although the provision of some signal functions (individually) was relatively higher in some countries, we observed major gaps when reporting the overall capacity (provision of all signal functions together). This emphasises the need for dropping vertical interventions and promoting a health system-strengthening approach focussed on primary healthcare related to reproductive, maternal, and child health services in these countries and other LMICs with similar challenges. The provision of communication with referral facilities and emergency transportation was also inadequate in most countries, except Afghanistan. Therefore, communication and transportation should be considered as a fundamental and priority component of the health system strengthening for enhancing primary healthcare.

Referral facilities serve as the final point of contact for patients requiring critical attention and specialised care after experiencing an abortion. These facilities should have comprehensive availability, readiness, and functionality of PAC services to deal with emergencies and complications, and they thus play a defining role in averting abortion-related deaths and disabilities [35]. We found that the overall capacity to provide comprehensive PAC services in referral facilities is somewhat better than the capacity to provide basic PAC services through primary facilities, but that it is still unacceptably inadequate, as it ranges between 52% (in DRC) and 16% (in Bangladesh) in the selected countries. Such gaps are also evident in providing comprehensive obstetric care [32–34] and paediatric inpatient care [36,37] in these selected countries and other resource-limited settings. The gaps are particularly prominent for performing blood transfusions and major surgeries, including caesarean sections. This delineates the under-preparedness of the referral facilities in managing medical emergencies and advanced complications related to reproductive, maternal and child health. Investing in referral hospitals to enhance the capacity of emergency management and perform major surgeries can substantially benefit reproductive health services, including comprehensive PAC services, and accelerate the progress towards achieving the SDG goals [38]. There are wide variations in the status of health facilities to provide basic and comprehensive PAC services based on individual signal functions, both within and across countries.

Afghanistan has one of the highest maternal mortality ratios globally [29], with unsafe abortion being one of the leading causes of maternal death [39]. However, the status of many of the PAC signal functions in Afghanistan is better than that of other countries included in our analysis. One of the possible explanations for such a high level of signal function provision (compared to other countries) is the operational definition of primary facilities. Only private clinics were included as primary facilities in Afghanistan’s SPA [40]. It is understandable that the availability, readiness and functionality of for-profit private facilities offering childbirth services could be better than the publicly funded primary care centres of other LMICs included in this analysis. Moreover, only 67 primary facilities were included from Afghanistan, which is considerably less than the other countries (206 in Bangladesh, 702 in Nepal, 193 in Ethiopia, and 296 in Haiti). Given this limited sample size and focus on private facilities, we are not certain whether this led to an overestimation or underestimation of the national capacity, but it may have affected the national representation of our findings for Afghanistan in any case. This high level of signal function provision may not translate into high-quality care due to gaps in the knowledge and capacity of birth attendants in both public and private facilities, as highlighted by previous research [29]. Prioritising reproductive and maternal health services and focussing on referral hospitals with significant technical assistance and financial investments by the global health and development communities might explain this high level of provision in Afghanistan, a country impacted by war, with predominantly conservative religious and social values [41,42]. However, this focus is often driven by specific donor interests, which may not adequately address the broader maternal health issues contributing to the high maternal mortality ratio [43,44].

Ethiopia, like Afghanistan, has a higher status for many of the signal functions needed to provide both basic and comprehensive PAC services compared to other countries. The provision is almost universal (close to 90% or more) for all basic and comprehensive signal functions, except communicating with referral facilities. In particular, Ethiopia is the only country in our analysis demonstrating more than 90% provision of modern short-acting family planning methods in both primary and referral facilities. These findings are consistent with another study reporting high (89%) capacity of health facilities to provide post-abortion family planning services [30]. In recent years, the Government of Ethiopia has taken a comprehensive approach to strengthen reproductive healthcare, including enhancing the post-abortion family planning services [45].

In Bangladesh, there wide variations in the status of health facilities to provide basic and comprehensive PAC services based on individual signal functions. Among basic signal functions, the provision was 98% for administering parenteral antibiotics and offering modern short-acting family planning methods. Conversely, the provision was particularly poor for communicating with referral facilities (8%) and emergency transportation (13%). There were also substantial gaps in the provision of removing retained products of conception (39%) and administering intravenous fluid (45%). The apparently high provision of administering parenteral antibiotics could be due to the wide availability, relatively inexpensive, and often indiscriminate use of antibiotics in Bangladesh [46]. Studies conducted in primary-level hospitals in the country found that 86–100% of hospitalised patients received antibiotics, predominantly based on empirical practices rather than following standard guidelines [47–49]. The Ministry of Health in Bangladesh has a dedicated directorate (known as the Directorate General of Family Planning) to promote and provide family planning services, which overlooks a large (>3500) network of primary facilities (known as Union Health and Family Welfare Centres) throughout the country [50]. Such Centres accounted for most of the primary facilities of Bangladesh included in our analysis. The relatively high provision of offering modern short-acting family planning methods could be due to the success of Bangladesh's vertical family planning programme and dedicated service delivery mechanism. However, such provision was not observed for communicating with referral facilities and emergency transportation. Other studies have also highlighted the critical gaps in the provision of referral care pathways in Bangladesh [51–53]. Although the clinical guidelines of Bangladesh acknowledge referral as a fundamental component of care, it is rarely prioritised in policy and programme planning. This is evident by the lack of strategic investments in emergency management, referral and transportation in the 4th Health, Population and Nutrition Sector Programme of Bangladesh, which was implemented between 2016 and 2024 [54]. This is also true for other comprehensive signal functions, such as blood transfusion and undertaking major surgeries, including caesarean section in referral facilities in Bangladesh.

Nepal, one of the South Asian countries, has demonstrated remarkable achievements in maternal and child health over the past two decades [55]. However, the status of most of the signal functions in Nepal was worse than that of other countries included in our analysis. In 2015, Nepal adopted a new constitution with three levels of self-governance: federal, provincial, and local/municipal. Seven provincial governments are now mandated to provide basic hospital services, in collaboration with local and federal authorities [56]. However, this transition from a centralised health system to a more decentralised governance structure has encountered various challenges resulting in significant gaps in the provision and coordination of several services. The sub-optimum provision of signal functions to provide basic and comprehensive PAC services could be linked to these unintended consequences of this transition. Also, a prior study reported significant gaps in service availability, supplies and human resources related to contraceptive counselling [57].

Upon closer examination of trends, most countries have shown slight improvements over the last decade, with Bangladesh demonstrating a decrease at the primary level. All countries have generally seen an increase, albeit minimal, in their ability to provide comprehensive PAC. Specifically, Senegal and Haiti observed significant progress in basic PAC is primary level facilities. This advancement in Senegal is attributed to reproductive health-focussed policies, increased international funding, and improved healthcare provider training [58]. Public awareness campaigns have also promoted care-seeking among women, aligning with broader initiatives aimed at enhancing maternal health and reducing unsafe abortion. In Haiti, international aid has bolstered training programmes and strengthened healthcare facilities [59]. Despite persistent political instability, these interventions, alongside community engagement and educational campaigns, have raised awareness of reproductive health and expanded PAC access for women [60]. In contrast, PAC capacity in Bangladesh has declined, mainly due to less effective health policies and shifting funding priorities away from maternal health [61,62]. Sociocultural barriers (such as abortion-related stigma) and limited healthcare infrastructure further restrict access to PAC services [22,63]. Addressing these barriers is essential to improving maternal health outcomes in Bangladesh.

Therefore, it remains important to highlight that proactive efforts are needed in line with SDG targets 3.7 and 5.6 of goals 3 and 5, respectively. These targets aim to achieve universal health coverage by ensuring access to essential sexual and reproductive healthcare services, while promoting gender equality through the empowerment of women and their reproductive rights.

### Strengths and limitations

We used SPA data sets from seven countries for this analysis. The SPA uses validated tools, adopts a standard data collection and data quality monitoring approach, and conducts surveys with nationally representative samples of health facilities. Therefore, we consider the validity, reliability (compared across countries and time), and representativeness of the findings to be among the main strengths of our study. Also, we used the signal functions, primarily developed by Campbell et al. [10] and later used by others [64,65], to report the overall capacity of health facilities to provide basic and comprehensive PAC services. Signal functions are a globally accepted and widely used approach to assess the capacity of health facilities, which has been in use in maternal health for the past few decades and has recently been gaining traction in research on newborn health [37,66,67]. We also reported the changes in the capacity to provide PAC services over time, offering insights into progress and areas needing improvement.

Our study also has several limitations. We reported the capacity to provide PAC services only among facilities offering childbirth services, but could not include those that do not due to the lack of information on key PAC signal functions. We are aware that some of the excluded facilities could have been offering PAC services. However, more than half of the referral facilities of the selected countries offer childbirth services, so we expect the effect of the possible selection bias on the overall capacity (reported in this paper) of facilities would be minimal. Yet admittedly, our estimates suggest that a large proportion (53%) of primary facilities in the selected countries do not offer childbirth services. We therefore acknowledge that the overall capacity of primary facilities reported here could be affected by sampling limitations, which may reduce the generalisability of our findings. While the SPA survey adopts a multi-stage sampling approach to ensure representative data, certain countries may have excluded specific types of health facilities from the sampling frame. This exclusion could affect the comprehensiveness of data, as the absence of particular facility types in countries like Afghanistan might lead to an incomplete reflection of health service capacity in those countries.

Usually, a facility is considered to have a signal function if it was performed in the past three months [68]. However, Campbell et al. [10] extended the period to 12 months to ever (perform the function) for several signal functions and included availability and readiness as criteria for defining some of the other signal functions. Although we used the operational definitions of signal functions proposed by Campbell et al. [10], we recommend interpreting the findings considering these differences. Lastly, we reported the change over time in the capacity of health facilities to provide basic and comprehensive PAC services. Although we observed some changes for several countries, most of these differences were not statistically significant, primarily due to the small sample size of health facilities included in our analysis.

## CONCLUSIONS

We observed substantial gaps in the capacity to deliver both basic and comprehensive PAC services in the selected countries. Among the basic signal functions, key challenges include inadequate communication with referral facilities and insufficient emergency transportation systems. To address these, it is essential to adopt a health system strengthening approach that prioritises the integration of reproductive, maternal, and child health services within primary healthcare frameworks, with particular emphasis on improving communication and transportation infrastructures. The gaps among comprehensive signal functions are particularly prominent for performing blood transfusion and major surgeries, including caesarean sections. Strengthening the capacity of referral hospitals to effectively manage obstetric emergencies and perform major surgeries can substantially increase the capacity of PAC services. Each country requires a context-specific investment strategy, informed by the gaps identified in this analysis (Table S8 in the **Online Supplementary Document**).

## Additional material


Online Supplementary Document
