# Supplementary material for: Assessing health systems’ capacities to provide post-abortion care: insights from seven low- and middle-income countries
Source: J Glob Health. 2025 Jan 10;15:04020. doi: 10.7189/jogh.15.04020 (PMC11719741; doi:10.7189/jogh.15.04020)
Supplement: Online Supplementary Document [file jogh-15-04020-s001.pdf]

Supplement to: Raza S, Banik R, Noor STA, Jahan E, Sayeed A, Huq N, Arifeen SE, Ahmed A, Rahman AE. Assessing health systems' capacities to provide post-abortion care: insights from seven low- and middle-income countries. J Glob Health. 2025;15:04020.

(A)

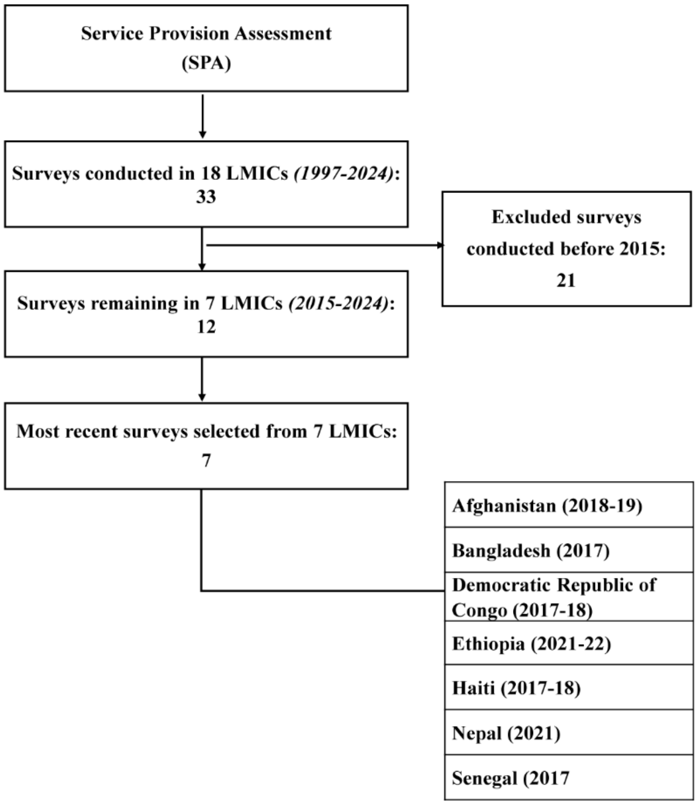

(B)

Country	Survey rounds	Latest round
Afghanistan	2018-19	2018-19
Bangladesh	2017	2017
Democratic Republic of Congo	2017-18	2017-18
Ethiopia	2021-22	2021-22
Haiti	2017-18	2017-18
Nepal	2015, 2021	2021
Senegal	2015, 2016, 2017, 2018, 2019	2019

Figure S1. Availability of Service Provision Assessment (SPA) Surveys Conducted Across Countries from 2015 to 2024.

Table S1. Distribution of the surveyed data used in the final analysis

Service Provision Assessment (SPA) Survey year	Year	Total			Primary				Referral			
		Completed survey (Weighted)	Completed survey & provide deliveries (weighted)		Completed survey (Weighted)		Completed survey & provide deliveries (weighted)		Completed survey (Weighted)		Completed survey & provide deliveries (weighted)	
		n	%	n	%	n	%	n	%	n	%	n
Afghanistan	2018-19	142	78.73	112	0.54	76	87.51	67	0.46	66	68.52	45
Bangladesh	2017	1524	23.51	358	95.59	1061	25.07	266	94.49	463	19.87	92
Nepal	2021	1576	51.07	805	0.90	1415	49.60	702	0.10	161	63.90	103
Democratic Republic of Congo	2017-18	1380	96.23	1328	0.90	1244	96.14	1196	0.10	136	97.49	132
Ethiopia	2021-22	1158	18.74	217	0.98	1134	17.03	193	0.02	24	98.27	24
Senegal	2019	425	57.11	243	0.97	412	56.09	231	0.03	13	89.76	12
Haiti	2017-18	1007	35.85	361	0.92	922	32.10	296	0.08	85	76.50	65

* Completed interviews are those where the facility has completed all four components of the SPA survey: a facility inventory questionnaire, client-provider observation protocols, client exit questionnaires and health worker questionnaires.

Table S2. Signal functions for basic and comprehensive post-abortion care [1]

**Capacity to provide both basic and comprehensive post-abortion care**<br>Expected functions for all facilities	
1) Remove retained products of conception*<br>2) Administer parenteral antibiotics*<br>3) Administer parenteral uterotonics*<br>4) Administer intravenous fluids†<br>5) Provide at least one modern, short-acting family planning method at the time of the survey†	
**Capacity to provide basic post-abortion care**<br>Functions expected of primary-level health facilities	**Capacity to provide comprehensive post-abortion care**<br>Functions expected of referral-level health facilities
6) Communicate with referral facilities†<br>7) Has a vehicle with fuel to transport patients needing referral†<br>8) Has staff capable of undertaking normal deliveries on duty or who are on call for 24 hours per day, 7 days per week	6) Administer a blood transfusion*<br>7) Undertake major abdominal surgery (proxied by the provision of caesarean section) *<br>8) Provide at least one long-acting, reversible family planning method† or permanent method at the time of the survey<br>9) Has staff capable of doing caesarean sections on duty or who are on call 24 hours per day, 7 days per week‡

*Assessed on the basis of facility reporting if they had ever provided the service. †Assessed on the basis of the availability and validity or functionality of a given item (drug or equipment) at the time of the survey. ‡We assumed that staff who were capable of doing caesarean sections were also capable of doing normal deliveries, and therefore did not include this factor in comprehensive capacity.

Table S3. Classification of primary and referral level health facilities across seven LMICs

Country	Facilities classified as primary	Facilities classified as referral
**Afghanistan**	▪ Private clinic	▪ Regional/ national hospital<br>▪ Provincial hospital<br>▪ Special hospital<br>▪ Private hospital
**Bangladesh**	▪ Community clinic<br>▪ Union subcenter/ rural dispensary<br>▪ Union Health and Family Welfare Center<br>▪ NGO clinic	▪ Upazila Health Complex District Hospital<br>▪ Private Hospital (with >20 beds)<br>▪ NGO Hospital<br>▪ Maternal and Child Welfare Center
**Nepal**	▪ Primary Health Care Center (PHCC)<br>▪ Health Post (HP)<br>▪ Sub-Health Post (SHP)<br>▪ Urban Health Center HTC (stand-alone)	▪ Central Government Hospital Regional<br>▪ Government Hospital Sub-Regional<br>▪ Government Hospital Zonal<br>▪ Government Hospital District<br>▪ Government Hospital Other Public Hospital<br>▪ Other Hospitals (not state-owned)
**Democratic Republic of Congo**	▪<br>▪ Primary Health Care Center<br>▪ Health Center	▪ Tertiary / Provincial Hospital<br>▪ General Referral Hospital<br>▪ Hospital / Hospital Center / Clinic
**Ethiopia**	▪ Health Center<br>▪ Health Post<br>▪ Higher Clinic<br>▪ Medium Clinic<br>▪ Lower Clinic<br>▪ Specialty Clinic	▪ Referral Hospital<br>▪ General Hospital<br>▪ Primary Hospital
**Senegal**	▪ Health Centre<br>▪ Health post<br>▪ Case de Sante (Health hut)	▪ Hospital
**Haiti**	▪ Dispensary<br>▪ Health Center without Lit<br>▪ Health Center with Lit Community Referral Hospital	▪ University Hospital Regional Hospital

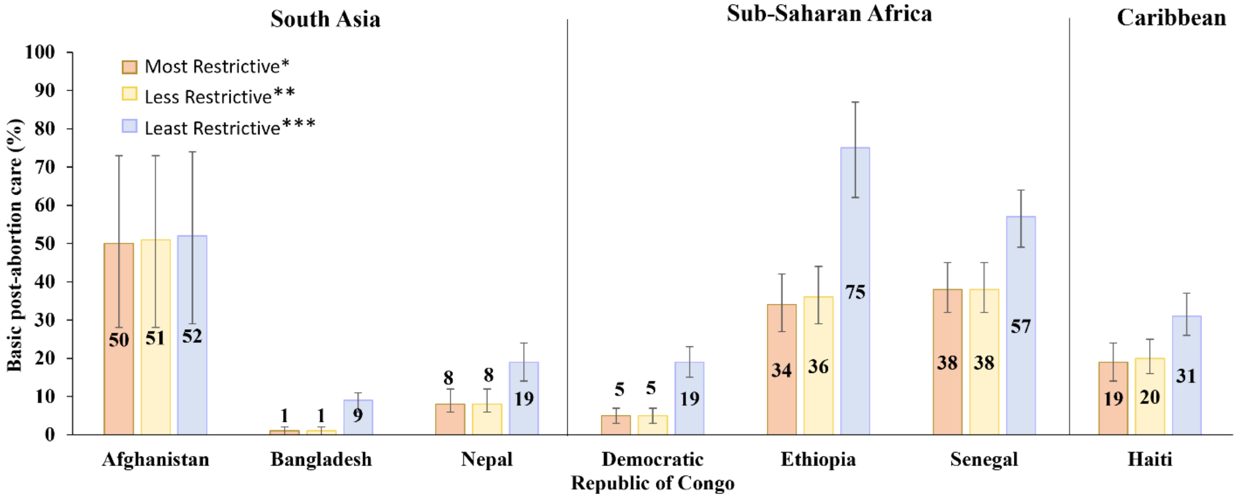

*All indicators. **Excluding staff with delivery ability for 24 h per day, 7 days per week. ***Excluding staff with delivery ability for 24 h per day, 7 days per week, and referral ability, including availability of short-acting, long-acting, or permanent family planning methods

Figure S2. Basic PAC capacity among primary-level facilities offering delivery services in seven LMICs, 2015–2024

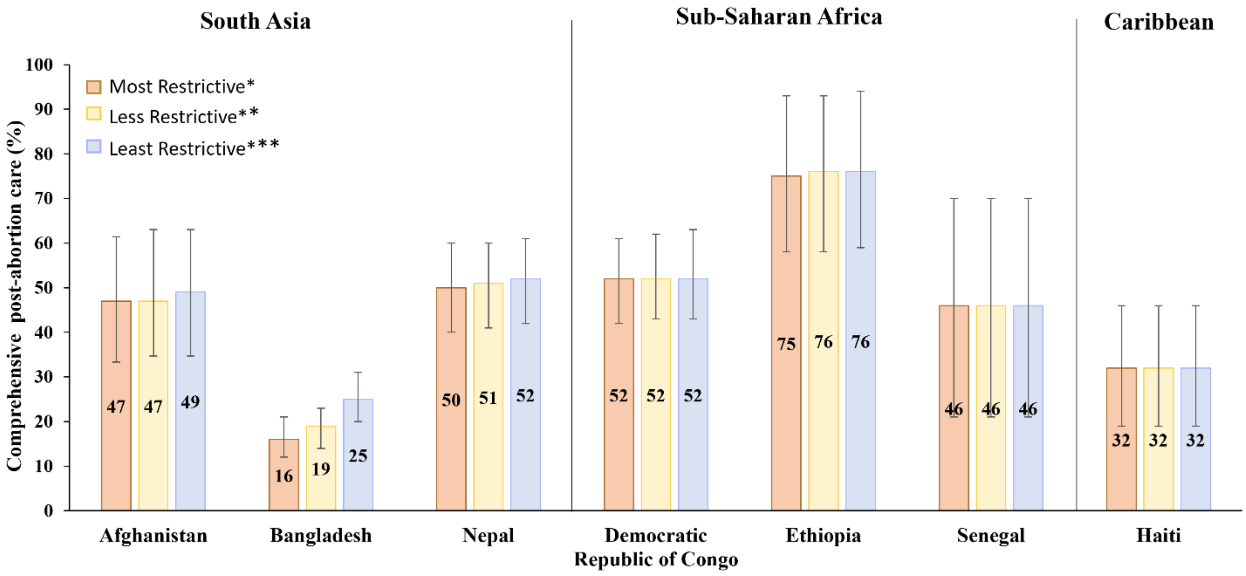

*All indicators. **Excluding staff with caesarean ability for 24 h per day, 7 days per week. ***Excluding ability to undertake major abdominal surgery and staff with caesarean ability for 24 h per day, 7 days per week.

Figure S3. Comprehensive PAC capacity among referral-level facilities offering delivery services in seven LMICs, 2015–2024

Table S4. Distribution of basic and comprehensive PAC capacity by facility type

Variables	Afghanistan			Bangladesh			Nepal		
	**Primary level**	**Referral level**	**Total**	**Primary level**	**Primary level**	**Total**	**Primary level**	**Referral level**	**Total**
	**% (CI)**	**% (CI)**	**% (CI)**	**% (CI)**	**% (CI)**	**% (CI)**	**% (CI)**	**% (CI)**	**% (CI)**
Basic PAC Capacity									
Most restrictive (all indicators)	50 (28, 73)	68 (56, 79)	58 (42, 72)	1 (0, 3)	43 (36, 50)	12 (9, 14)	5 (3, 7)	25 (19, 32)	7 (5, 9)
Less restrictive (excluding staff with delivery ability for 24 hours per day, 7 days per week)	51 (28, 73)	71 (59, 81)	59 (44, 73)	1 (0, 3)	44 (37, 51)	12 (9, 15)	5 (3, 7)	25 (19, 32)	7 (5, 9)
Least restrictive (excluding staff with delivery ability for 24 hours per day, 7 days per week, and referral capacity; including availability of short-acting, long-acting, or permanent family planning methods)	52 (29, 74)	65 (52, 76)	57 (42, 71)	9 (6, 11)	43 (36, 50)	17 (11, 25)	19 (16, 23)	52 (46, 59)	23 (20, 26)
Comprehensive PAC Capacity									
Most restrictive (all indicators)	48 (26, 71)	47 (35, 60)	48 (33, 63)	0.2 (0, 1)	16 (12, 21)	4 (3, 6)	6 (5, 9)	52 (45, 58)	11 (9, 13)
Less restrictive (excluding staff with caesarean ability for 24 hours per day, 7 days per week)	49 (27, 72)	47 (35, 60)	48 (34, 64)	0.2 (0, 1)	19 (14, 23)	5 (4, 6)	6 (5, 9)	52 (45, 59)	11 (9, 13)
Least restrictive (excluding ability to undertake major abdominal surgery and staff with caesarean ability for 24 h per day, 7 days per week)	49 (27, 72)	49 (37, 61)	49 (34, 64)	0.2 (0, 1)	25 (20, 31)	7 (5, 8)	8 (6, 10)	52 (46, 59)	12 (10, 15)

Table S4 (Continued). Distribution of PAC capacity by facility type

Variables	Democratic Republic of Congo			Ethiopia			Senegal			Haiti		
	Primary level	Referral level	Total	Primary level	Referral level	Total	Primary level	Referral level	Total	Primary level	Referral level	Total
	% (CI)	% (CI)	% (CI)	% (CI)	% (CI)	% (CI)	% (CI)	% (CI)	% (CI)	% (CI)	% (CI)	% (CI)
Basic PAC Capacity												
Most restrictive (all indicators)	5 (3, 7)	25 (19, 32)	7 (5, 9)	34 (27, 42)	78 (72, 83)	39 (33, 46)	38 (32, 45)	62 (39, 81)	39 (33, 46)	19 (15, 24)	29 (19, 41)	21 (17, 25)
Less restrictive (excluding staff with delivery ability for 24 hours per day, 7 days per week)	5 (3, 7)	25 (19, 32)	7 (5, 9)	36 (29, 44)	79 (73, 84)	41 (34, 48)	38 (32, 45)	62 (39, 81)	39 (33, 46)	20 (16, 25)	29 (19, 41)	22 (18, 26)
Least restrictive (excluding staff with delivery ability for 24 hours per day, 7 days per week, and referral capacity; including availability of short-acting, long-acting, or permanent family planning methods)	19 (16, 23)	52 (46, 59)	23 (20, 26)	75 (68, 81)	87 (83, 91)	76 (70, 81)	57 (49, 64)	62 (39, 81)	57 (50, 64)	31 (26, 37)	38 (27, 51)	32 (28, 37)
Comprehensive PAC Capacity												
Most restrictive (all indicators)	6 (5, 9)	52 (45, 58)	11 (9, 13)	0 (0, 1)	75 (69, 81)	9 (7, 11)	1 (0, 2)	46 (25, 67)	3 (2, 5)	6 (4, 10)	32 (22, 44)	11 (8, 15)
Less restrictive (excluding staff with caesarean ability for 24 hours per day, 7 days per week)	6 (5, 9)	52 (45, 59)	11 (9, 13)	0 (0, 1)	76 (69, 81)	9 (7, 11)	1 (0, 2)	46 (25, 67)	3 (2, 5)	8 (5, 11)	32 (22, 44)	12 (9, 16)
Least restrictive (excluding ability to undertake major abdominal surgery and staff with caesarean ability for 24 h per day, 7 days per week)	8 (6, 10)	52 (46, 59)	12 (10, 15)	0 (0, 1)	76 (70, 82)	9 (7, 11)	2 (1, 4)	46 (25, 67)	4 (3, 6)	9 (6, 13)	32 (22, 44)	13 (10, 17)

Table S5. Availability of basic and comprehensive PAC signal functions among facilities that offer delivery services in seven LMICs

Variables	Afghanistan			Bangladesh			Nepal		
	**Primary level**	**Referral level**	**Total**	**Primary level**	**Referral level**	**Total**	**Primary level**	**Referral level**	**Total**
	**% (CI)**	**% (CI)**	**% (CI)**	**% (CI)**	**% (CI)**	**% (CI)**	**% (CI)**	**% (CI)**	**% (CI)**
Removal of retained products of conception	84 (54, 96)	97 (87, 99)	89 (70, 97)	39 (33, 47)	75 (69, 81)	49 (43, 55)	40 (35, 46)	79 (71, 85)	45 (40, 50)
Administer parenteral antibiotics	84 (61, 94)	92 (82, 97)	87 (74, 94)	29 (23, 36)	93 (89, 95)	45 (40, 51)	53 (47, 59)	88 (78, 94)	58 (52, 63)
Administer parenteral uterotonics	94 (86, 98)	92 (82, 97)	93 (87, 97)	50 (42, 57)	97 (95, 98)	62 (56, 67)	95 (91, 97)	96 (88, 99)	95 (92, 97)
Administer intravenous fluids	100	100	100	45 (38, 53)	94 (91, 96)	58 (52, 64)	99 (98, 100)	99 (98, 100)	99 (98, 100)
Modern short-acting family planning methods available	80 (58, 92)	90 (79, 96)	84 (71, 92)	98 (95, 99)	74 (67, 81)	92 (89, 94)	100	89 (85, 93)	99 (98, 99)
Capacity to communicate with referral facilities	91 (62, 98)	98 (87, 100)	94 (76, 99)	8 (9, 19)	91 (83, 91)	29 (27, 37)	82 (77, 86)	98 (96, 99)	84 (80, 88)
Vehicle with fuel for referral	93 (84, 97)	95 (86, 99)	94 (88, 97)	13 (5, 13)	87 (86, 94)	32 (25, 34)	31 (26, 37)	89 (81, 94)	39 (34, 44)
Staff capable of undertaking vaginal deliveries available 24 hours per day, 7 days per week	95 (87, 98)	95 (87, 99)	95 (90, 98)	48 (40, 55)	95 (91, 98)	60 (54, 66)	98 (96, 99)	97 (88, 99)	98 (96, 99)
Administer a blood transfusion	75 (50, 90)	78 (66, 87)	76 (61, 86)	2 (1, 5)	54 (47, 61)	15 (13, 19)	0 (0, 2)	76 (68, 83)	10 (8, 12)
Undertake major abdominal surgery (proxied by provision of caesarean section)	84 (54, 96)	97 (87, 99)	89 (70, 97)	39 (33, 47)	75 (69, 81)	49 (43, 55)	0 (0, 0)	74 (66, 81)	10 (8, 11)
Provided at least one long-acting, reversible family planning method or the permanent method at the time of survey	84 (61, 94)	92 (82, 97)	87 (74, 94)	29 (23, 36)	93 (89, 95)	45 (40, 51)	66 (61, 72)	87 (81, 92)	69 (64, 74)
Has staff capable of doing caesarean sections on duty or who are on call 24 hours per day, 7 days per week	94 (86, 98)	92 (82, 97)	93 (87, 97)	50 (42, 57)	97 (95, 98)	62 (56, 67)	0 (0, 0)	73 (65, 80)	9 (8, 11)

Table S5 (Continued). Distribution of PAC capacity by facility type.

Variables					Ethiopia			Senegal			Haiti	
	Primary level	Referral level	Total	Primary level	Referral level	Total	Primary level	Referral level	Total	Primary level	Referral level	Total
	% (CI)	% (CI)	% (CI)	% (CI)	% (CI)	% (CI)	% (CI)	% (CI)	% (CI)	% (CI)	% (CI)	% (CI)
Removal of retained products of conception	63 (59,67)	82 (77,86)	65 (61,69)	86 (80,90)	97 (95,98)	87 (82,91)	74 (68,80)	87 (64,96)	75 (69,80)	71 (65,76)	82 (70,89)	73 (68,77)
Administer parenteral antibiotics	79 (75,82)	95 (92,97)	80 (77,83)	94 (90,96)	99 (98,100)	94 (91,97)	77 (71,82)	96 (77,99)	78 (72,83)	84 (79,88)	100	87 (83,90)
Administer parenteral uterotonics	96 (94,97)	99 (98,100)	96 (94,97)	99 (97,99)	99 (98,100)	99 (97,99)	95 (92,97)	100	96 (92,98)	94 (90,96)	98 (90,100)	94 (92,96)
Administer intravenous fluids	81 (77,84)	93 (88,96)	82 (79,85)	97 (93,98)	96 (94,98)	97 (93,98)	94 (90,96)	100	94 (90,96)	96 (93,98)	100	97 (94,98)
Modern short-acting family planning methods available	69 (65,73)	80 (73,86)	70 (66,74)	99 (96,100)	95 (93,97)	98 (96,99)	95 (83,99)	75 (50,90)	94 (84,98)	82 (77,86)	59 (46,70)	78 (73,82)
Capacity to communicate with referral facilities	16 (13,20)	53 (46,59)	20 (17,23)	88 (82,92)	100 (98,100)	89 (84,93)	70 (62,76)	96 (77,99)	71 (63,77)	47 (41,52)	66 (54,77)	50 (45,55)
Vehicle with fuel for referral	51 (47,55)	66 (60,72)	52 (48,56)	48 (40,56)	88 (84,91)	52 (45,59)	77 (71,82)	100	78 (72,83)	75 (69,79)	94 (85,98)	78 (74,82)
Staff capable of undertaking vaginal deliveries available 24 hours per day, 7 days per week	96 (94,97)	98 (93,99)	96 (94,97)	93 (87,96)	99 (98,100)	94 (89,97)	99 (95,100)	100	99 (95,100)	82 (77,86)	98 (90,100)	85 (81,88)
Administer a blood transfusion	28 (25,32)	98 (96,99)	35 (32,39)	1 (0,2)	87 (82,90)	10 (8,13)	4 (2,6)	79 (51,93)	7 (5,10)	19 (15,24)	88 (77,94)	31 (27,36)
Undertake major abdominal surgery (proxied by provision of caesarean section)	63 (59,67)	82 (77,86)	65 (61,69)	1 (0,2)	92 (89,95)	11 (9,13)	1 (1,3)	79 (51,93)	5 (3,8)	17 (13,22)	86 (75,93)	29 (25,34)
Provided at least one long-acting, reversible family planning method or the permanent method at the time of survey	79 (75,82)	95 (92,97)	80 (77,83)	93 (88,96)	98 (97,99)	93 (89,96)	99 (96,100)	100	99 (96,100)	62 (56,67)	84 (73,91)	66 (61,71)
Has staff capable of doing caesarean sections on duty or who are on call 24 hours per day, 7 days per week	96 (94,97)	99 (98,100)	96 (94,97)	1 (0,2)	91 (87,94)	11 (8,13)	1 (1,3)	79 (51,93)	5 (3,8)	15 (11,19)	81 (70,89)	27 (23,32)

Table S6. Distribution of facilities with delivery services by survey years.

Variables	Afghanistan	Bangladesh		Nepal		Democratic Republic of Congo	Ethiopia	Senegal					Haiti	
	2019	2014	2017	2015	2022	2018	2022	2015	2016	2017	2018	2019	2013	2018
No. of facilities with delivery services (n)	112	280	358	458	805	1328	217	363	355	477	267	243	389	361
Primary-level facilities (n)	67	200	266	438	702	1196	193	350	343	465	257	231	330	296
Referral-level facilities (n)	45	80	92	20	103	132	24	13	12	12	10	12	59	65
Basic PAC in primary-level facilities (%)	50	4	1	6	8	5	34	16	19	18	39	38	12	19
Comprehensive PAC in referral-level facilities (%)	47	8	16	34	50	32	75	32	51	45	58	46	25	32
P-value*		0.016		0.103				0.001					0.007	
P-value**		0.056		0.096				0.24					0.195	

***Proportion test of the capacity of providing basic PAC in primary-level facilities between the oldest and most recent survey**
***Proportion test of the capacity of providing comprehensive PAC in referral-level facilities between the oldest and most recent survey**

Table S7. Health and socioeconomic indicators in seven LMICs.

Country	Population (2023)	Population Density (per km²)	GDP (USD, 2023)	Health Expenditure (% of GDP)	Maternal Mortality Ratio (per 100,000 live births)	ANC Coverage (% of women with at least four visits)	Crude Birth Rate (per 1,000 people)	Total Fertility Rate (births per woman)	Contraceptive Rate (%)
Afghanistan	~42.2 million	~65	$14.5 billion	~11%	620	21%	35.1	4.3	16%
Bangladesh	~171.5 million	~1,169	$437.4 billion	~2.36%	123	47%	18.5	2.1	62%
Nepal	~30.9 million	~210	$37.1 billion	~5.17%	151	76%	19.9	1.8	50%
Democratic Republic of Congo	~102 million	~41	$60 billion	~3.79%	547	49%	40.5	6.11	20%
Ethiopia	~126 million	~112	$156 billion	~3.48%	267	62%	31.5	3.82	41%
Senegal	~18 million	~85	$28 billion	~4.35%	315	54%	33.1	4.34	23%
Haiti	~12 million	~419	$21 billion	~3.48%	529	45%	23.9	2.75	35%

Source: https://data.worldbank.org/

Table S8. Country-specific recommendation on PAC service capacity

Country	Recommendations
Afghanistan	Strengthen capabilities at referral facilities for blood transfusions to manage abortion-related emergencies.
Bangladesh	Prioritise an uninterrupted supply of essential commodities and medicines, strengthen communication and transportation systems, and ensure 24/7 staff availability at primary facilities. Additionally, bolster referral facilities with enhanced capabilities for blood transfusions, abdominal surgeries, and continuous staff availability for caesarean deliveries.
Nepal	Bolster PAC capacity by investing in reliable supply chains for essential commodities, establishing efficient communication and referral networks, and ensuring 24/7 staffing at primary facilities. Furthermore, enhance referral facilities with robust blood transfusion services, advanced surgical capabilities, and continuous staff availability for caesarean deliveries.
DRC	Improve vehicle availability and fuel supply at primary facilities to facilitate effective patient transportation for referrals, reduce delays in emergency care.
Haiti	Haiti must prioritise expanding the supply of modern short-acting family planning methods in referral facilities while ensuring functional transportation for referrals in primary facilities to bolster PAC service capacity.
Ethiopia	Strengthen communication capabilities within primary facilities to improve PAC service provision and timely coordination of care.
Senegal	Should work on increasing capacity for the removal of retained products of conception and improving vehicle availability and fuel supply at primary-level facilities.

References (Supplementary)

1 Campbell OMR, Aquino EML, Vwalika B, Gabrysch S. Signal functions for measuring the ability of health facilities to provide abortion services: An illustrative analysis using a health facility census in Zambia. *BMC Pregnancy Childbirth* 2016; **16**. DOI:10.1186/s12884-016-0872-5.
